# Association Between Hemorrhoids and Lower Extremity Chronic Venous Insufficiency

**DOI:** 10.7759/cureus.4502

**Published:** 2019-04-19

**Authors:** Ugur Ekici, Abdulcabbar Kartal, Murat F Ferhatoglu

**Affiliations:** 1 General Surgery, Istanbul Gelişim University, Istanbul, TUR; 2 General Surgery, Okan University Medical Faculty, Istanbul, TUR

**Keywords:** hemorrhoidal disease, varicose veins, ceap classification, chronic constipation

## Abstract

Aim

The aim of the present study was to evaluate the incidence of varicose veins among patients with hemorrhoidal disease and to compare its incidence reported in various community-based studies.

Method

The study group comprised of 100 patients who underwent surgery for symptomatic internal or external hemorrhoids; the control group consisted of 100 volunteers who received no prior therapy for hemorrhoidal disease and lacked any symptoms or findings suggestive of this condition. Subjects in both the groups were inquired with respect to their demographic data and risk factors. Both groups were asked to stand for two minutes before performing leg examinations while still in the standing position. The findings were recorded for both the groups. Varicose veins were classified according to the clinical appearance section of the Clinical, Etiologic, Anatomic, and Pathophysiologic (CEAP) classification that was developed by the 1994 American Venous Forum.

Results

There was no significant difference between the two groups with respect to age and body mass index (BMI). Significant relationships were identified between the groups with respect to the incidence of varicose veins and chronic constipation. The incidence of C1 and C2 varicose veins observed in the study group was higher than that observed in the control group. The incidence of chronic constipation was higher in the study group than that in the control group.

Discussion

Lower extremity chronic venous insufficiency is more common in patients with hemorrhoidal disease which increases intra-abdominal pressure. A chronic increase in this pressure causes conditions, such as constipation, which trigger both lower extremity chronic venous insufficiency and hemorrhoidal disease.

## Introduction

Hemorrhoidal disease is a very common condition that affects the anorectal area and is characterized by the distal displacement of the anal cushions, which results in associated symptoms [1]. The weakening of the suspensory ligaments in the third decade of life causes the anal cushions to sag and dilate toward the lumen. Consequently, the passage of stool exerts pressure on the anal cushions, leading to vascular congestion and bleeding. The addition of inflammation to this condition results in hemorrhoidal disease [2]. The risk factors of hemorrhoidal disease include conditions that generally increase intra-abdominal pressure, such as constipation, diarrhea, pregnancy, delivery, obesity, standing for extended time periods, and chronic coughing. Hemorrhoidal disease is most frequently observed in individuals aged 65-74 years, although its occurrence tends to decrease after 75 years of age. It is rarely observed in individuals aged >20 years. One cause of the condition is the decrease in muscle tone in the anal region that is associated with increasing age. However, studies conducted in the 1980s have demonstrated that the high incidence of this condition generally observed in the elderly has shifted toward middle-aged individuals, which has been interpreted as a consequence of changes in nutritional habits. The incidence of hemorrhoidal disease does not exhibit any gender variability and is similar in women and men according to age. In addition, the condition is neither associated with socioeconomic factor distribution nor familial predisposition. Although its incidence does not show any particular regional variability, it is slightly common among people living in rural areas [3]. Hospital-based proctoscopy studies show that although most patients are asymptomatic, its incidence can be as high as 86% [4].

Varicose veins are frequently encountered among adults. According to the Framingham Study, USA, the two-year incidence of varicose veins in women and men was 2.6% and 2.0%, respectively [5]. The incidence of varicose veins in the Western population is estimated to be 25%-30% among women and 10%-20% among men [6]. This condition is caused by the functional impairment of the one-way valves in the veins of the lower extremities that prevent the blood from flowing back under the effect of gravity, and this impairment can be either congenital or acquired. Varicose veins are an indication of lower extremity venous insufficiency. Such veins can appear superficial, expanded, elongated, and tortuous. They are generally asymptomatic in their early stages, while the most common symptoms of varicose veins are localized swelling, a sense of heaviness, cramps, pain, chronic localized weakness, itching, and formication. Varicose veins may lead to more severe symptoms such as superficial thrombophlebitis, bleeding, lipodermatosclerosis, skin hyperpigmentation, and venous ulcers [6]. The well-known risk factors of varicose veins are sex (more common among women), pregnancy, warm climate, age, weight, and a life style that requires the person to stand or sit for extended time periods.

Hemorrhoidal disease and lower extremity varicose disease have similar risk factors. The aim of the present study was to evaluate the incidence of varicose veins among patients with hemorrhoidal disease and to compare its incidence reported in various community-based studies.

## Materials and methods

The study group (Group 1) comprised of 100 patients who underwent surgery between March 2014 and March 2015 at the Malatya Public Hospital for Grade 3-4 symptomatic internal or external hemorrhoids. The control group (Group 2) consisted of a 100 volunteers who received no prior therapy for hemorrhoidal disease and lacked any symptoms or findings suggestive of this condition. Subjects in both the groups were inquired with respect to their demographic data and risk factors. On the morning of their surgery, patients in Group 1 were asked to stand for two minutes before performing leg examinations while still in the standing position. Patients in Group 2 underwent leg examinations after obtaining their medical histories with respect to hemorrhoidal disease and performing physical examinations. The findings were recorded for both the groups. Varicose veins were classified according to the clinical appearance section of the Clinical, Etiologic, Anatomic, and Pathophysiologic (CEAP) classification that was developed by the 1994 American Venous Forum (Table 1) [7]. Age, body mass index (BMI), sex, chronic diseases, chronic constipation and presence of varicose veins were compared between the groups.

**Table 1:** Varicose veins classified according to the Clinical, Etiologic, Anatomic, and Pathophysiologic (CEAP) classification

C0: No signs of venous disease
C1: Telangiectasias or reticular veins
C2: Varicose veins
C3: Edema
C4: Skin or subcutaneous skin changes
C5: Healed venous ulcer
C6: Active venous ulcer

Statistical methods

The Shapiro-Wilk test was used to assess the normality of the data. Variables with and without normal distribution were compared between two the groups using the Student’s t-test and Mann-Whitney U test, respectively. The relationships between two independent variables at a categorical measurement level were assessed using the Chi-square test. For descriptive statistics, categorical variables were expressed as number and percentage. Statistical analysis was performed using the SPSS Windows version 24.0 (IBM Corp., Armonk, NY) package software, and a p-value of < 0.05 was considered to be statistically significant.

## Results

The mean age and mean BMI of patients in both the groups were similar (Table 2). There was no statistically significant difference between the two groups with respect to these variables (p = 0.971 and p = 0.934­, respectively).

**Table 2:** Evaluation of qualitative variables within the groups

Variables	Patient (n = 100) Mean ± SD	Control (n = 100) Mean ± SD	T	p
Age	36.89 ± 12.3	36.83 ± 11.19	0.04	0.971
BMI	26.17 ± 3.95	26.22 ± 3.84	−0.08	0.934

Statistically significant relationships were identified between the study and control groups with respect to the incidence of varicose veins and chronic constipation (each, p < 0.05). As such, the proportion of patients without varicose veins (81%) in the control group was higher than that of these patients in the study group (65%). Moreover, the incidence of C1 and C2 varicose veins observed in the study group was significantly higher than that observed in the control group (p = 0.039).

Similarly, the incidence of chronic constipation was significantly higher in the study group (78.0%) than that in the control group (29.0%) (p < 0.001). No significant relationship was identified between the other evaluated variables and the groups (p > 0.05) (Table 3).

**Table 3:** Evaluation of qualitative characteristics within the groups

		Group					
		Study (n = 100)		Control (n = 100)			
		n	%	n	%	x^2 ^	p
Presence of varicose veins	C0	65	65.0	81	81.0	6.497	0.039
	C1	20	20.0	11	11.0		
	C2	15	15.0	8	8.0		
Sex	Male	55	55.0	52	52.0	0.181	0.671
	Female	45	45.0	48	48.0		
Pregnancy/delivery	Yes	6	10.9	6	11.5	0.011	0.918
	No	49	89.1	46	88.5		
Working in sitting position for long time periods	Yes	16	16.0	16	16.0	0.001	1.000
	No	84	84.0	84	84.0		
Standing for long time periods	Yes	51	51.0	45	45.0	0.721	0.396
	No	49	49.0	55	55.0		
Chronic diseases	Yes	19	19.0	17	17.0	0.136	0.713
	No	81	81.0	83	83.0		
Chronic Constipation	Yes	78	78.0	29	29.0	48.256	0.001
	No	22	22.0	71	71.0		

## Discussion

Lower extremity venous insufficiency and hemorrhoidal disease are two clinical conditions that are commonly observed and negatively affect the quality of life of the patients. Chronic venous insufficiency can occur as a result of primary and secondary causes (70% and 30% of cases, respectively) and usually presents comorbidities, such as venous hypertension, which are independent of its etiology. It is often associated with underlying modifiable (smoking, obesity, pregnancy, standing, straining during defecation and constipation, and deep vein thrombosis) or non-modifiable (female sex and genetic predisposition) risk factors. In most cases, hemorrhoidal disease stems from causes associated with an increase in the intra-abdominal pressure (constipation, pregnancy, ascites, obesity, chronic coughing, and prostatism). A chronic increase in this pressure can lead to additional pressure/load on the muscles and venous system in the anorectal area, causing the pooling of blood in the venous plexus, which lacks valves. The types and ranges of comorbidities that accompany hemorrhoids suggest that the pathophysiological mechanism responsible for the development of hemorrhoidal disease includes an increase in the anal sphincter tone and chronic venous hypertension [8]. Lower extremity venous disease is a common problem and it can result in significant morbidity and mortality. The final common pathway that leads to chronic venous insufficiency is the development of venous hypertension, particularly within the deep venous system [9].

The data of the patients, such as those related to age, height, and weight, were similar between Group 1 and Group 2 (p = 0.971 and p = 0.934, respectively). These data are important for ensuring that the patients in the study and control groups have similar demographic characteristics.

There was a significant association between the two groups in terms of the incidence of varicose veins and chronic constipation (p < 0.05). Particularly, the incidence of C1 and C2 varicose veins was significantly higher in the study group than that in the control group (35% and 19%, respectively; p = 0.039). This higher incidence in Group 1 suggests that hemorrhoidal disease and lower extremity chronic venous insufficiency may be caused by similar etiological factors. Determining the incidence of telangiectasias and reticular varicose veins was difficult due to the absence of corresponding data in many studies and also due to the difference in the classification of these conditions. The Edinburgh Vein Study determined that the incidence of reticular varicose veins and telangiectasias was >80% and that the majority of identified cases had a relatively mild form of these conditions. The same study determined that the incidence of trunk varicose veins was 20% among men and 5.3% among women between the ages of 18 and 24 years, whereas it was 61.4% among men and 50.5% among women between the ages of 55 and 64 years [5].

In the present study, the incidence of chronic constipation was significantly higher in the study group (78.0%) than that in the control group (29.0%) (p < 0.001). Other variables such as sex, the number of births, working in a sitting or standing position for extended time periods, and chronic diseases were similar between the two groups (p > 0.05). These findings were considered significant for demonstrating that chronic constipation may be a common cause of both the conditions. Chronic constipation is one of the most frequently observed digestive problems, and its incidence in society may be far greater than that assumed. The estimated prevalence of chronic constipation in North America varies between 2% and 27% [10]. The study by Arora G et al. determined that chronic constipation is associated with many clinical conditions such as gastroesophageal reflux, anal fissures, anal fistula, diverticulosis, and colon cancer but particularly with hemorrhoidal disease [11]. Kartal A et al. published a study and demonstrated that chronic constipation has an impact on the development of inguinal hernia [12]. Our literature review revealed no studies demonstrating a direct relationship between constipation and lower extremity venous insufficiency. However, the frequent occurrence of hemorrhoidal disease with varicose veins suggests that chronic constipation may be an underlying cause of lower extremity venous diseases.

## Conclusions

Our prospective observational study demonstrated that lower extremity chronic venous insufficiency is more common in patients with hemorrhoidal disease which increases intra-abdominal pressure. A chronic increase in this pressure causes conditions, such as constipation, which trigger both lower extremity chronic venous insufficiency and hemorrhoidal disease. Future studies with larger case series will further highlight this topic.
